# Supplementary material for: Sea Urchin-like Si@MnO2@rGO as Anodes for High-Performance Lithium-Ion Batteries
Source: Nanomaterials (Basel). 2022 Jan 17;12(2):285. doi: 10.3390/nano12020285 (PMC8778068; doi:10.3390/nano12020285)
Supplement: Supplementary file 1 [file nanomaterials-12-00285-s001.zip › nanomaterials-1559350-supplementary.pdf]

nanomaterials

Supplementary Materials

Sea Urchin-Like Si@MnO2@rGO as Anodes for High-Performance Lithium-Ion Batteries

Jiajun Liu, Meng Wang, Qi Wang, Xishan Zhao, Yutong Song, Tianming Zhao and Jing Sun *

College of Sciences, Northeastern University, Shenyang 110819, China; 2000165@stu.neu.edu.cn (J.L.); 2000176@stu.neu.edu.cn (M.W.); wangqi@mail.neu.edu.cn (Q.W.); 2000189@stu.neu.edu.cn (X.Z.); 2000172@stu.neu.edu.cn (Y.S.); zhaotm@stumail.neu.edu.cn (T.Z.).

* Correspondence: sunjing74@mail.neu.edu.cn; Tel.: +86 136-0407-3045

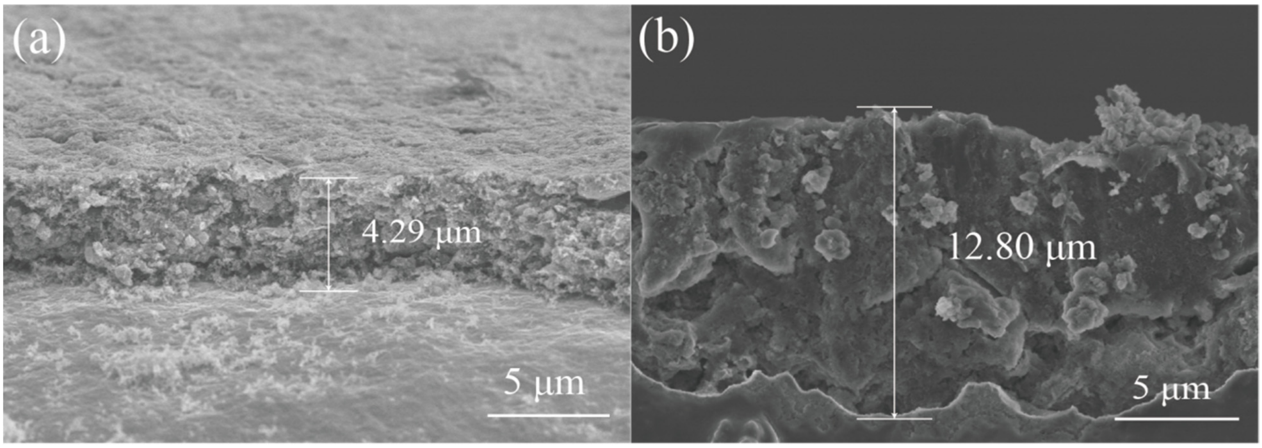

Figure S1. (**a**,**b**) Cross-sectional SEM images of Si@MnO_2-50℃ before and after 150 cycles.

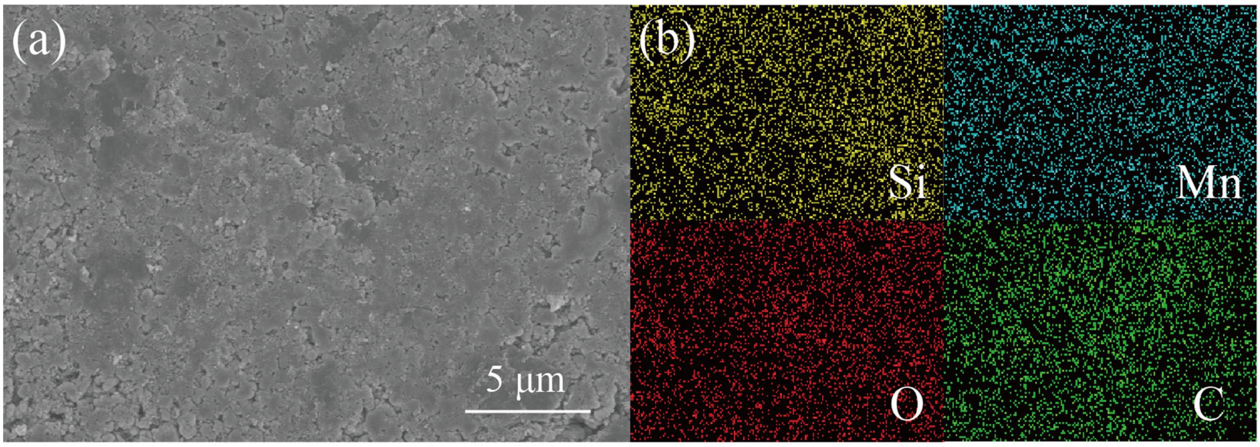

Figure S2. (**a**) Cross-sectional SEM images of the Si@MnO_2@rGO-50℃ electrode sheet after 150 cycles at 0.1 A g^{-1}. (**b**) Element mapping images of the Si@MnO_2@rGO-50℃ electrode sheet after 150 cycles at 0.1 A g^{-1}.

Table S1. Capacity contribution of Si, MnO_2 and rGO.

Sample	Theoretical Capacity	Atom Percentage	Weight Percentage	Capacity Contribution
Si	4200 mAh g^{-1}	47%	32%	63.3%
MnO_2	1223 mAh g^{-1}	31%	62%	35.6%
rGO	372 mAh g^{-1}	21%	6%	1.1%

Table S2. Synthesis strategies and electrochemical performance comparison Si-based anode materials and MnO_2-based anode materials in lithium-ion batteries.

Sample	Synthesis Method	Cycle Retention	Cycling Stability (mAh/g)	Refs.
Si@MnO_2@rGO	Stirring and freeze-dry	88% after 1000 cycles at 1A g^{-1}	1282 mAh g^{-1} after 1000 cycles at 1A g^{-1}	This work
Porous Si/rGO	Stirring, chemical etching and water bath.	75% after 200 cycles at 1 A g^{-1}	About 1026mAh g^{-1} after 50 cycles at 1 A g^{-1}	[61]
Si/rGO	Stirring, freeze-dry and thermal treatment	53% after 200 cycles at 1 A g^{-1}	About 550mAh g^{-1} after 200 cycles at 1 A g^{-1}	[20]
Si/rGO	Stirring, chemical etching, calcine and ball-milling	67% after 300 cycles at 0.5 A g^{-1}	548 mAh g^{-1} after 300 cycles at 0.5 A g^{-1}	[46]
CL-Si@C/rGO	Calcine and stirring	About 50% after 100 cycles at 1 A g^{-1}	910 mAh g^{-1} fter 100 cycles at 1 A g^{-1}	[62]
α-Fe_2O_3/MnO_2	Calcine and stirring	About 62% after 500 cycles at 0.5 A g^{-1}	494 mAh g^{-1} after 500 cycles at 0.5 A g^{-1}	[63]
MnO_2/rGO	Hydrothermal method	About 44% after 500 cycles at 0.5 A g^{-1}	About 500 mAh g^{-1} after 500 cycles at 0.5 A g^{-1}	[64]

Table S3. The R_S, and R_{CT} values fitted from the equivalent circuit model are summarized for comparison.

Electrode	R_S/Ω	R_{CT}/Ω
Si	5.8	272.1
Si@MnO_2	3.8	233.0
Si@MnO_2@rGO	11.3	146.2
Si@MnO_2@rGO-150th	13.7	72.7
